# High Rate of Virological Response to Peginterferon α-2a–Ribavirin Among Non-Cirrhotic Iranian Hemophilia Patients With Chronic Hepatitis C

**Published:** 2012-08-30

**Authors:** Mostafa Alavi Moghaddam, Mohammad Reza Zali, Seyed Hossein Aalaei Andabili, Faramarz Derakhshan, Seyed Mohammad Miri, Seyed Moayed Alavian

**Affiliations:** 1 Research Institute of Gastroenterology and Liver Diseases, Shahid Beheshti University of Medical Sciences, Tehran, IR Iran; 2 Baqiyatallah University of Medical Sciences, Baqiyatallah Research Center for Gastroenterology and Liver Disease (BRCGL), Tehran, IR Iran

**Keywords:** Hepatitis C, Hemophilia A, Hemophilia B, Peginterferon alfa-2a, Ribavirin, Iran

## Abstract

**Background:**

Hepatitis C is a major reason of morbidity and mortality among hemophilia patients. Although combination therapy with peginterferon (peg-INF) and ribavirin is considered as standard treatment for chronic hepatitis C (CHC), but more evidence of the efficacy and safety is needed.

**Objectives:**

In this study, efficacy and tolerability of combination therapy with peginterferon α-2a–ribavirin was investigated among hemophilia HCV infected patients.

**Patients and Materials:**

In a quasi-experimental, 45 naive hemophilia patients with chronic HCV received 180 mg of pegylated interferon (Pegasys) by subcutaneous injection weekly plus an oral dose of 800-1200 µg ribavirin daily according to body weight. The treatment continued 48 weeks in patients with genotype one and 24 weeks in those with genotype 3. Sustained virological response (SVR) was considered as efficacy of treatment.

**Result:**

Forty three patients (95.6%) reached to end of treatment response (ETR); only two (4.4%) patients did not respond and were discontinued from treatment. None of 43 patients relapsed. SVR obtained in 43 of 45 patients (95.6%), in multivariate logistic regression model, third month’s treatment WBC (WBC > 2000) remained the only significant predictor of SVR. Regimen dose reduced in three patients; two of those because of ALT increasing and other one for his retinal bleeding. In repeated measurement analysis, alanine aminotransferase (ALT) and hemoglobin (Hb) decreased significantly during treatment, but reduction of platelet (PLT) was not significant.

**Conclusons:**

Results show high efficacy and safety of combination therapy of Peg-IFN-α 2a plus ribavirin among hemophiliacs with chronic hepatitis C.

## 1. Background

Approximately 200 million individuals are infected with hepatitis C virus (HCV) worldwide [1]. HCV is responsible for 70% of all chronic hepatitis patients, 40% of all cases of liver cirrhosis, 60% of hepatocellular carcinomas (HCC), and 30% of liver transplants in Europe [2]. In addition, HCV is recognized as the leading indication for liver transplantation and cause of 8,000–10,000 deaths annually [3]. All hemophiliacs who have been transfused with clotting factor concentrates before 1985 and/or blood transfusions before 1992 have been exposed to the HCV and approximately 100% of these are positive for HCV antibody. HCV prevalence among patients with hemophilia is variable from 24% to 95% in different parts of the world [2][4][5]. However, it is conservatively estimated from 15 to 80% in various parts of Iran [6][7]. The most common genotype between hemophilia HCV infected patients is 1a followed by 3a [8]. Combination therapy with (peginterferon) peg-IFN and ribavirin is considered as standard treatment for chronic hepatitis C (CHC). In addition, peginterferon (alfa-2a and alfa-2b) are two approved and available forms of pegylted INF. Two systematic reviews, one with 12 RCTs and another with 7 RCTs reported advantage of peginterferon α-2a over 2b [9][10]. To the best of our knowledge, there are not enough data regarding the treatment of HCV-infected coagulation disorders with peginterferon α-2a on Caucasian subjects [11].

## 2. Objectives

The objective of this study is to evaluate the response of HCV infected hemophilia patients to combination antiviral therapy with peginterferon α-2a plus ribavirin.

## 3. Patients and Materials

### 3.1. Patients and Study Design

This was prospective, single-centre, open-label one-arm treatment study of peginterferon α- 2a (Pegasyss, Roche) and ribavirin (Copeguss, Roche) combination therapy of chronic hepatitis C infection in non-cirrhotic subjects with hemophilia. Exclusion criteria comprised decompensated liver disease, hepatitis B virus or HIV co-infection, liver dysfunction, liver transplants, HCC, creatinine clearance < 50 ml/min, severe psychiatric illness, uncontrolled diabetes, severe cardiac or chronic pulmonary diseases, malignant neoplastic diseases, autoimmune diseases, substance abuse within one year before entry, and retinopathy. All eligible subjects of study were treated with 180 µg of Pegasys by subcutaneous injections once weekly plus an oral dose of 800-1200 mg ribavirin per day according to body weight. Patients with HCV genotype 1 with a weight less than 75 kg received 1000 mg of oral ribavirin daily, while patients over 75 kg were given 1200 mg of oral ribavirin per day. Patients with genotype non-1 were treated with a daily 800 mg dose of ribavirin. The treatment continued for 48 weeks in patients with HCV genotype 1 and for 24 weeks in those with genotype 3. All subjects provided informed consent, and the protocol was approved by the Ministry of Health appointed protocol review board.

### 3.2. Assessment of Efficacy

HCV RNA titers were measured at baseline, 12, 24, and 48th week during the treatment phase and 24 weeks of untreated follow up. The primary end point for efficacy was a sustained virological response (SVR), defined as undetectable HCV-RNA in serum after 24 weeks of untreated follow up. The secondary end point was early viral response (EVR: Undetectable HCV RNA in 12th week of treatment) and end of treatment response (ETR: Undetectable HCV RNA at the end of the treatment). Patients with an inadequate virological response at 12 weeks or at 24 weeks of treatment were considered as treatment failure and treatment was discontinued.

### 3.3. Assessment of Safety

Safety was assessed by laboratory tests monthly and evaluation of adverse events during treatment and follow up periods. Adverse events were graded as mild, moderate, and severe or life-threatening with a modified world health organization (WHO) grading system. The laboratory criteria for decreasing of peginterferon α-2a dose were 500–750 neutrophils/mm3 or 30000–50000 platelets (PLT)/mm^3^ Also, reduction of the neutrophil count to less than 500 cells/mm^3^, PLT count to less than 30000/mm^3^, and hemoglobin (Hb) to less than 7 g/dl were considered as criteria for peginterferon discontinuation. The ribavirin dose was decreased when the Hb level dropped below 10 g/dl and was discontinued when it dropped below 8.5 g/ml.

### 3.4. Virological Methods

HCV genotyping was performed as described previously [12]. Quantification of viral load was carried out in a single laboratory using the cobas amplicor HCV monitor, v2.0 (Roche Diagnostics, Branchburg, NJ, USA) with a lower HCV RNA detection level of 50 IU/ml.

### 3.5. Statistical Analysis

The data were analyzed with SPSS version 17. Categorical variables were expressed as frequency and percentage. Univariate analysis was used to calculate odds ratio and then, multivariate logistic regression analysis was performed on all variables P < 0.2 to identify the independent predictors in the SVR, and to calculate adjusted odds ratio. Repeated measurement was used for determination of alanine aminotransferase (ALT), Hb, and PLT changes during treatment and comparison of their levels before and after treatment.

## 4. Results 

### 4.1. Patient’s Characteristics

The quasi-experimental study included 45 naive hemophilia patients who were chronically HCV-mono-infected. Majority were male whit hemophilia A. The mean of patient’s viral load was 2419174.96 (SD ± 6691582.995) IU/ml and genotype 1 was the most common (55.6%) type that followed by genotype 3 (42.2%), there was only one untypable genotype (2.2%). The mean age of patients was 30.4 (SD ± 12.6) (range: 13-76) years.

### 4.2. Efficacy

Early virological response (EVR) was achieved in 41 out of 45 patients (91.1%), end of treatment response (ETR) was seen in 43 of 45 patients (95.6%) and SVR obtained in all of 43 (95.6%) patients with ETR, and only two (4.4%) patients did not respond and were discontinued from the treatment. In the univariate analysis, there was not any significant predictor of a SVR. In multivariate logistic regression analysis, the third month of treatment WBC (WBC > 2000) remained the only significant predictor of SVR with odd ratio (OR): 42 (95% CI: 1.3-128.7). No other association was found.

### 4.3. Safety

Peginterferon α-2a and ribavirin dose was decreased in 3 (6.6%) patients respectively, two of them because of ALT increasing during treatment and another because of retinal bleeding. The most common side effects were leucopenia in 21 (46.6%), depression in 8 (17.7%), and weight loss > 5% in 4 (8.8%) patients.

### 4.4. ALT, PLT and Hb Changes During Treatment in Patients With SVR

**Figure 1:**
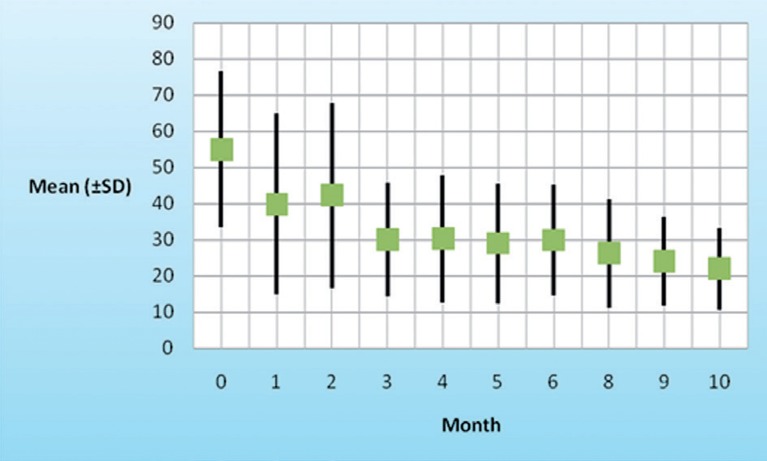
ALT Mean Reduction During Treatment

**Figure 2:**
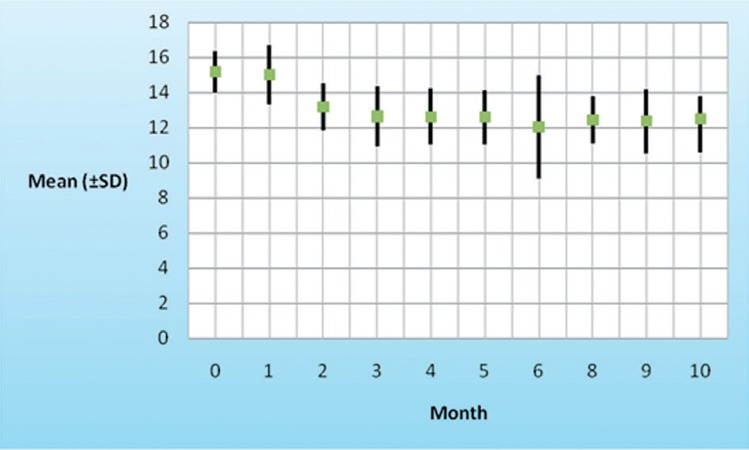
Hb Mean Reduction During Treatment Period

According to the repeated measurement analysis (means of ALT, PLT, and Hb in baseline, first-6th and 8-10th month of the study period and comparison with baseline levels), mean of ALT levels after beginning of treatment decreased (P = 0.001) from 55 (SD ± 21.5) to 21.9 (SD ± 11.4), and major reduction was in the first month of treatment (Figure 1). Reduction in platelet counts from baseline were observed transiently and sever reduction of PLT was occurred in two first weeks of treatment cession, but not significant. Hb decreased (P = 0.01) during treatment from baseline and a fall in Hb to less than 10 g/dl occurred in one (2.3%) patient (Figure 2).

## 5. Discussion

This study shows effectiveness and safety of Peg-IFNα-2a and ribavirin combination therapy. There was an association with third month of treatment WBC > 2000 (border of severe leucopenia) and SVR in our study, while other previously reported associations remained insignificant in this report [13][14][15][16]. We think other factors are involved in hypo-responsiveness of patients without SVR. Better IL28B polymorphism among study subjects (CC &TT versus TT & GG) [17] , young and non-cirrhotic patients, and genotype 3 which was common among them may have leaded to high rate of response rate. The study confirms high SVR rate (95%) in Iranian patients as Alavian reported (61%) [18], versus European patients (SVR rate of 63%) that is higher than American population (SVR rate of 45%). Lower rate of SVR in US may be obtained from various ethnicity and environmental related factors [19][20][21]. Our study included patients from Tehran (capital of Iran) and likely with Homogeneous ethnicity, environments, and even tolerability. There were only genotypes 1a and 3a. Genotype 1a is the most common in Iranian hemophilia patients [8][18]. One study [2], with its large sample size compared treatment out come between sub-genotypes of 1a and 1b. They found difficult response to treatment in patients with sub-genotype 1b. We found no significant difference between present genotypes of our study because of our small sample size and high SVR rate. We could compare genotypes, and find possible significant differences between their SVR rates if there were more patients. In the present study, peginterferon α-2a and ribavirin dose was decreased in (3/45) 6.6% of the patients respectively. While the rate of treatment withdrew was 0%. Perhaps because of lower age of our patients versus other studies, and the characteristics of hemophilia patients, who are compliant with HCV treatment because they are accustomed to comply with the treatment for their chronic and prolonged disease [2][14][15][19][22][23]. Treatment with peginterferon induced decreasing of RBC and PLT during treatment period. Because of our good treatment follow up, discontinuing of treatment regimen was not needed [14][24]. We found 17% depression near to previous report from Iran with 19% depression [2]. Rate of this side effect was 8% in Mancuso’s report between European patients [14]. While according to Fried’s study, depression rate is more in American patients, and approximately 20% to 30% of patients treated with peg-interferon and ribavirin have had this experience [22]. Depression occurrence has been reported less in treated patients with peginterferon and ribavirin versus treatment with standard interferon and ribavirin in Fried’s study [25]. Despite these adverse events, none of them was uncontrollable and life threatening. In conclusion, treatment with Peg-IFNα-2a and ribavirin is safe and effective especially among Iranian hemophilia HCV infected patients.
